# A Potential Anti-Glioblastoma Compound LH20 Induces Apoptosis and Arrest of Human Glioblastoma Cells via CDK4/6 Inhibition

**DOI:** 10.3390/molecules28135047

**Published:** 2023-06-28

**Authors:** Yan Wang, Youbin Li, Dong Liu, Danyang Zheng, Xiaogang Li, Chang Li, Caihui Huang, Yun Wang, Xuesong Wang, Qifu Li, Junyu Xu

**Affiliations:** 1 Key Laboratory of Tropical Translational Medicine of Ministry of Education, Hainan Key Laboratory for Research and Development of Tropical Herbs, Haikou Key Laboratory of Li Nationality Medicine, School of Pharmacy, Department of Neurology, The First Affiliated Hospital of Hainan Medical University, Hainan Medical University, Haikou 571199, China; hy0207116@hainmc.edu.cn (Y.W.); liyoubinli@sohu.com (Y.L.); liudong990122@163.com (D.L.); zhengdy2335@163.com (D.Z.); llg178701425@163.com (X.L.); lichang211270@163.com (C.L.); d603618327@163.com (C.H.); yunwang8899@163.com (Y.W.); wxuesong88@126.com (X.W.); 2 Key Laboratory of Brain Science Research & Transformation in Tropical Environment of Hainan Province, Hainan Medical University, Haikou 571100, China

**Keywords:** LH20, glioblastoma, apoptosis, G2/M, mitochondrial, CDKs

## Abstract

Glioblastoma (GBM) is a deadly brain tumor characterized by signaling dysregulation and aberrant cell cycle control. The CDK4/6-Rb axis is dysregulated in approximately 80% of all GBM cases. In this study, the anti-GBM effect of a novel pyrimidin-2-amine, LH20 was evaluated in vitro using the primary GBM cell lines U87MG and U251. GBM cells were administered LH20 at concentrations of 0.1, 1, 4, 8, 10, 20, 100, and 200 µM for 24 and 48 h, and the proliferation rate was evaluated using a CCK8 assay. Migration, apoptosis, and cell cycle were also assessed using a wound healing assay, Annexin V-FITC/PI apoptosis assay, and cell cycle staining, respectively. The targets of LH20 were predicted using SwissTargetPrediction and molecular docking. Western blotting analysis was performed to confirm the anti-GBM mechanism of LH20. We found that at concentrations of 4, 8, and 10 µM, LH20 significantly inhibited the proliferation and migration of U87MG and U251 cells, induced late phase apoptosis, promoted tumor cell necrosis, and arrested the G2/M phase of the cell cycle. LH20 also inhibited CDK4 and CDK6 activities by decreasing the phosphorylation of Rb. Our results suggest LH20 as a potential treatment strategy against GBM.

## 1. Introduction

Glioblastoma multiforme (GBM) is the most common and lethal malignant brain tumor with high molecular heterogeneity, poor overall prognosis, and a meager 10-year survival rate of <1%; only 6.9% of patients survive for more than 5 years following diagnosis [1]. Current standard first-line therapeutic strategies for GBM treatment include surgical resection of the primary tumor followed by radiotherapy and adjuvant temozolomide (TMZ), while other alkylating agents including lomustine and carmustine, and the anti-angiogenic agent bevacizumab, have been approved for the treatment of recurrent GBM [2]. However, the therapy for progressive or relapsed GBM remains limited [3]. Therefore, drugs with high efficacy for treating GBM are urgently needed to improve patient survival and prognosis.

Cell cycle dysregulation leads to cell cycle arrest, apoptosis, and cancer [4]. The hallmarks of genomic instability in GBM are perturbations in the cell cycle, including in the G1, S-phase, and G2/M transition [5]. Cyclin-dependent kinases (CDKs) are vital cell cycle checkpoints. Under physiological conditions, activation of CDK4 and CDK6 induces the G1 phase to initiate DNA synthesis, whereas that of CDK2 by cyclin A2 drives the transition from the S phase to mitosis, i.e., G2 phase. At the end of the interphase, CDK1 is activated to facilitate the onset of mitosis [6]. Genomic profiling has defined GBM subgroups and identified alterations in the core signaling pathways that are associated with CDK, such as CDKN2A/B, TP53, PTEN, EGRF amplification, CDK amplification, and CDK6 amplification [7,8]. Moreover, molecular analyses have shown that the CDK4/6-Rb axis is dysregulated in approximately 80% of GBM cases [9]; therefore, the inhibition of CDKs may be a promising treatment strategy against GBM.

To date, several CDK inhibitors have been approved by the FDA for the treatment of advanced-stage or metastatic, hormone-receptor-positive, HER2-negative breast cancers [10,11], including palbociclib, ribociclib, and abemaciclib (Abe). However, no CDK inhibitors have been approved for the treatment of GBM. Recent studies have shown that combined treatment with the CDK4/6 inhibitor Abe and an oncolytic virus significantly suppresses GBM tumor growth and prolongs the survival of tumor-bearing mice [12]. These findings suggest that targeting CDK4/6 could potentially inhibit GBM.

This study aimed to explore the anti-GBM effects of LH20, a novel pyrimidin-2-amine compound similar to Abe, in U87MG and U251 cell lines. Our findings will provide novel insights into the treatment of GBM, which will help to improve patients’ survival and quality of life.

## 2. Results

### 2.1. LH20 Inhibits GBM Cell Proliferation

**Figure 1:**
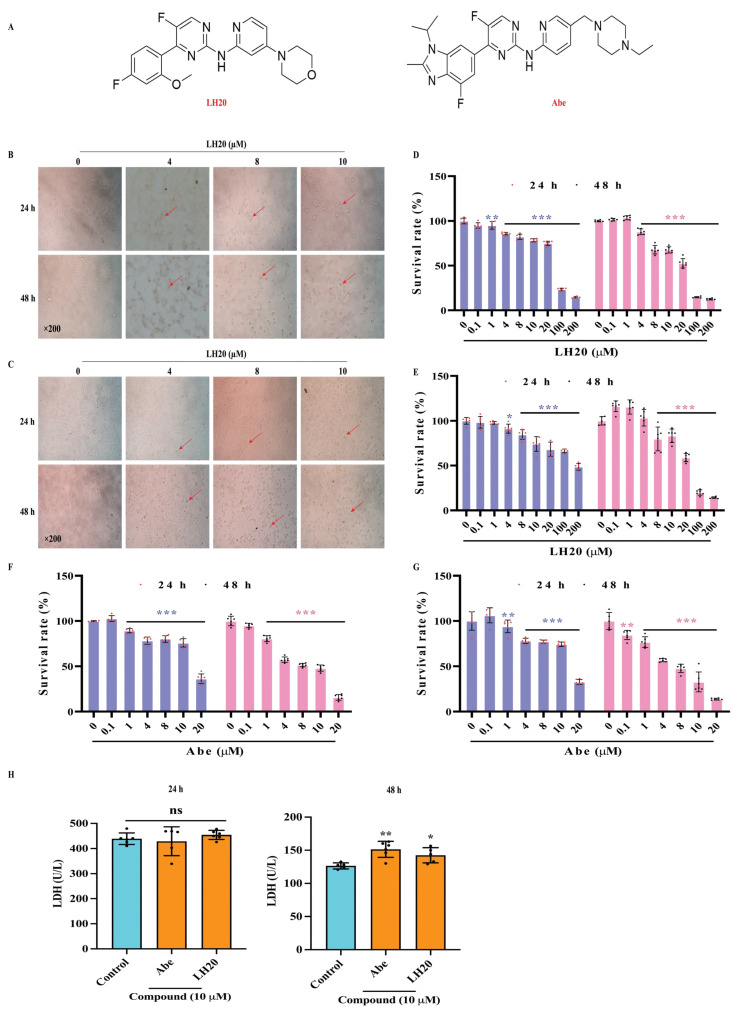
Effects of LH20 on the survival of GBM cells. (**A**) Compound structure of LH20 and Abe. (**B**) Morphological and cell density variation in U87MG cells following treatment for 24 h (**top**) and 48 h (**bottom**) with different concentrations of LH20. (**C**) Morphological and cell density variation in U251 cells observed following treatment for 24 h (**top**) and 48 h (**bottom**) with different concentrations of LH20. Red arrows represented the U87MG and U251 cells exhibited morphological changes, including cell rounding and the disappearance of protruding spikes (**D**) Effects of LH20 on U87MG cell proliferation using a CCK-8 assay (24 h and 48 h). (**E**) Effects of LH20 on U251 cell proliferation (24 h and 48 h). (**F**) Effects of Abe on U87MG cell proliferation (24 h and 48 h). (**G**) Effects of Abe on U251 cell proliferation (24 h and 48 h). (**H**) Effects of LH20 (10 μM) and Abe (10 μM) on the LDH release in U87MG cells. Data are expressed as mean ± standard deviation; ns represented no significance, * *p* < 0.05, ** *p* < 0.01, *** *p* < 0.001 compared with the untreated or control group.

U87MG and U251 cells treated with LH20 (Figure 1A) exhibited morphological changes, including cell rounding and the disappearance of protruding spikes (Figure 1B,C). The CCK8 assay showed that treatment with LH20 at 4, 8, 10, 20, 100, and 200 μM for 24 h significantly inhibited the survival of U87MG (survival rate ranged from 85.85 ± 1.38% to 14.86 ± 0.84%) and U251 (from 89.62 ± 3.55% to 48.70 ± 3.75%), compared with the control group (U87MG: 100.00 ± 3.18%, U251: 100.00 ± 3.85%).

The inhibitory activity of LH20 at 4, 8, 10, 20, 100, and 200 μM was augmented when cells were treated for 48 h (U87MG: from 87.95 ± 3.25% to 12.58 ± 0.87% vs. 100.00 ± 0.96%; U251: (8, 10, 20, 100, and 200 μM: from 79.83 ± 13.25% to 14.21 ± 0.95% vs. 100.00 ± 4.75%, Figure 1D,E). Similar anti-GBM effects were observed following treatment with Abe, which suppressed the survival of GBM cells at the concentrations of 1–20 μM for 24 h (Figure 1F) and 0.1–20 μM for 48 h (Figure 1G).

Once cells undergo injury or apoptosis, the membranes are disrupted and lactate dehydrogenase (LDH) is released into the culture medium. In this study, treatment with LH20 and Abe at 10 μM for 48 h significantly increased the LDH release from U87MG cells (Figure 1H), suggesting that prolonged administration of LH20 could induce injury to U87MG.

### 2.2. LH20 Suppresses GBM Cell Migration

**Figure 2:**
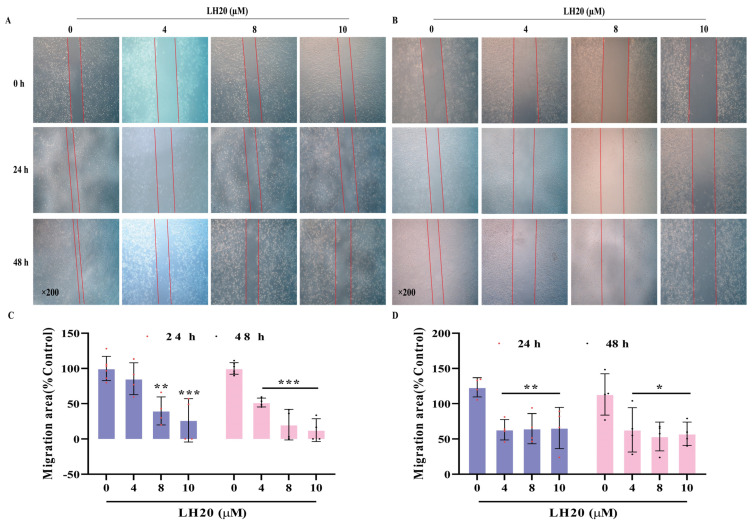
Effect of LH20 on the migration of U87MG and U251 cells. The migratory potential of GBM cells was analyzed using a wound healing assay. U87MG (**A**) and U251 (**B**) cells were incubated in the absence or presence of LH20 (4, 8, and 10 μM) for 48 h. Cells that migrated into the gap were counted under an optical microscope. Red lines indicate the edge of the gap. (**C**) Migratory area analysis of U87MG cells treated with LH20 for 24 h and 48 h. (**D**) Migratory area analysis of U251 cells treated with LH20 for 24 h and 48 h. Data are expressed as mean ± standard deviation; * *p* < 0.05, ** *p* < 0.01, *** *p* < 0.001 compared with the untreated group.

Scratched cells cultured in high-glucose Dulbecco’s Modified Eagle Medium (DMEM) for 24 and 48 h showed apparent migration (Figure 2A,B). U87MG cells treated with LH20 at 8 and 10 μM for 24 h significantly inhibited the migration area of cells (39.84 ± 19.86% and 26.50 ± 30.78%, respectively) compared with the control U87MG cells (100.00 ± 17.02%). The migration area further decreased in U87MG cells treated with LH20 at 4, 8, and 10 μM for 48 h (51.76 ± 6.26%, 20.22 ± 21.67%, and 12.58 ± 16.15% vs. 100.00 ± 8.25%, respectively; Figure 2C). Similar suppression effects were observed in LH20-treated U251 cells (24 h: 63.03 ± 14.52%, 64.63 ± 21.40%, 65.52 ± 29.31% vs. 123.30 ± 12.57%; 48 h: 62.98 ± 31.45%, 53.56 ± 20.31%, 57.36 ± 16.57% vs. 113.2 ± 29.24%, Figure 2D), suggesting that LH20 could suppress the migration of GBM cells.

### 2.3. LH20 Induces GBM Cell Apoptosis and Mitochondria Membrane Potential (MMP) Drop

**Figure 3:**
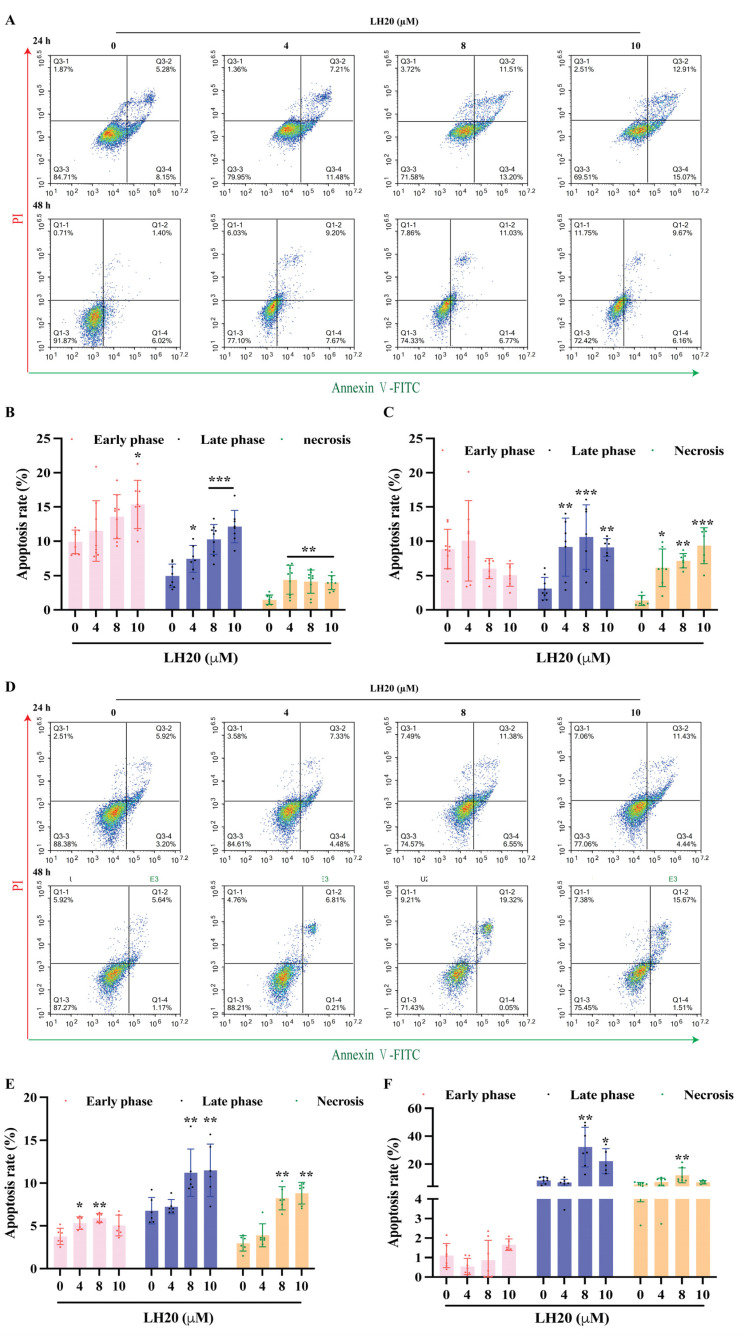
LH20 induced apoptosis in GBM cells. (**A**) Representative graphs from flow cytometry showing U87MG cells. Changes in early apoptosis, late apoptosis, and necrosis rates of U87MG cells treated with LH20 for 24 h (**B**) and 48 h (**C**). (**D**) Representative graphs from flow cytometry showing U251 cells. Changes in early apoptosis, late apoptosis, and necrosis rates of U251 cells treated with LH20 for 24 h (**E**) and 48 h (**F**). * *p* < 0.05, ** *p* < 0.01, *** *p* < 0.001 compared with the untreated group.

To evaluate the effects of LH20 on cell apoptosis, U87MG and U251 cells were treated with LH20 at 4, 8, and 10 μM for 24 or 48 h and assessed with Annexin Ⅴ-fluorescein isothiocyanate (FITC)/propidium iodide (PI). The U87MG cells treated with 8 and 10 μM LH20 exhibited higher late apoptosis (24 h: 10.26 ± 2.19% and 12.13 ± 2.36% vs. 4.97 ± 1.71%; 48 h: 10.61 ± 4.70% and 9.09 ± 1.30% vs. 3.12 ± 1.63%) and necrosis rates (24 h: 4.14 ± 1.71% and 3.99 ± 1.02% vs. 1.47 ± 0.62%; 48 h: 7.16 ± 1.05% and 9.36 ± 2.61% vs. 1.37 ± 0.65%, Figure 3A–C). Similar effects of LH20 were observed in U251 cells (Figure 3D–F).

**Figure 4:**
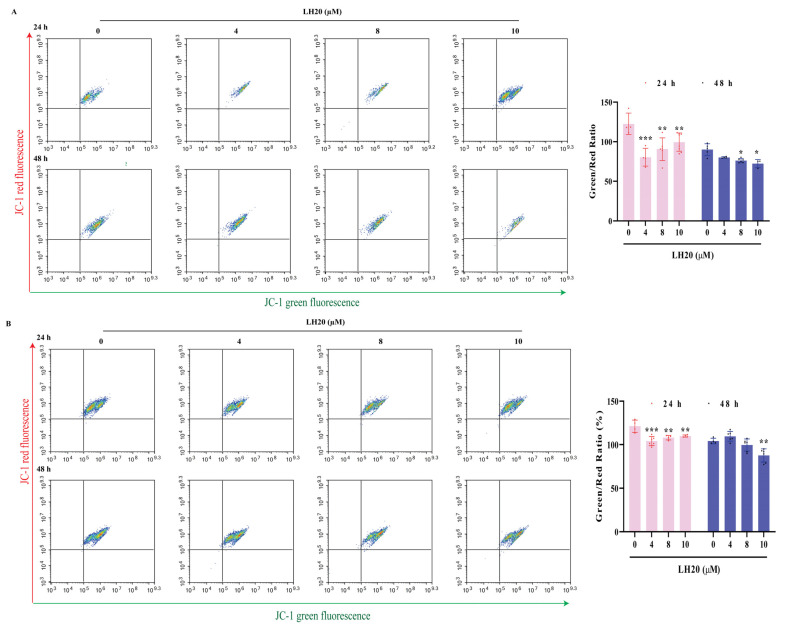
LH20 induced MMP drop in U87MG (**A**) and U251 cells (**B**). * *p* < 0.05, ** *p* < 0.01, *** *p* < 0.001 compared with the untreated group.

A JC-1 kit was used to detect the MMP of GBM cells. U87MG (Figure 4A) and U251 cells (Figure 4B) treated with LH20 for 24 or 48 h had a lower green/red ratio, suggesting that LH20 reduced the mitochondrial electron potential and induced mitochondrial-dependent apoptosis.

### 2.4. LH20 Arrested the GBM Cell Cycle at G2/M Phase

**Figure 5:**
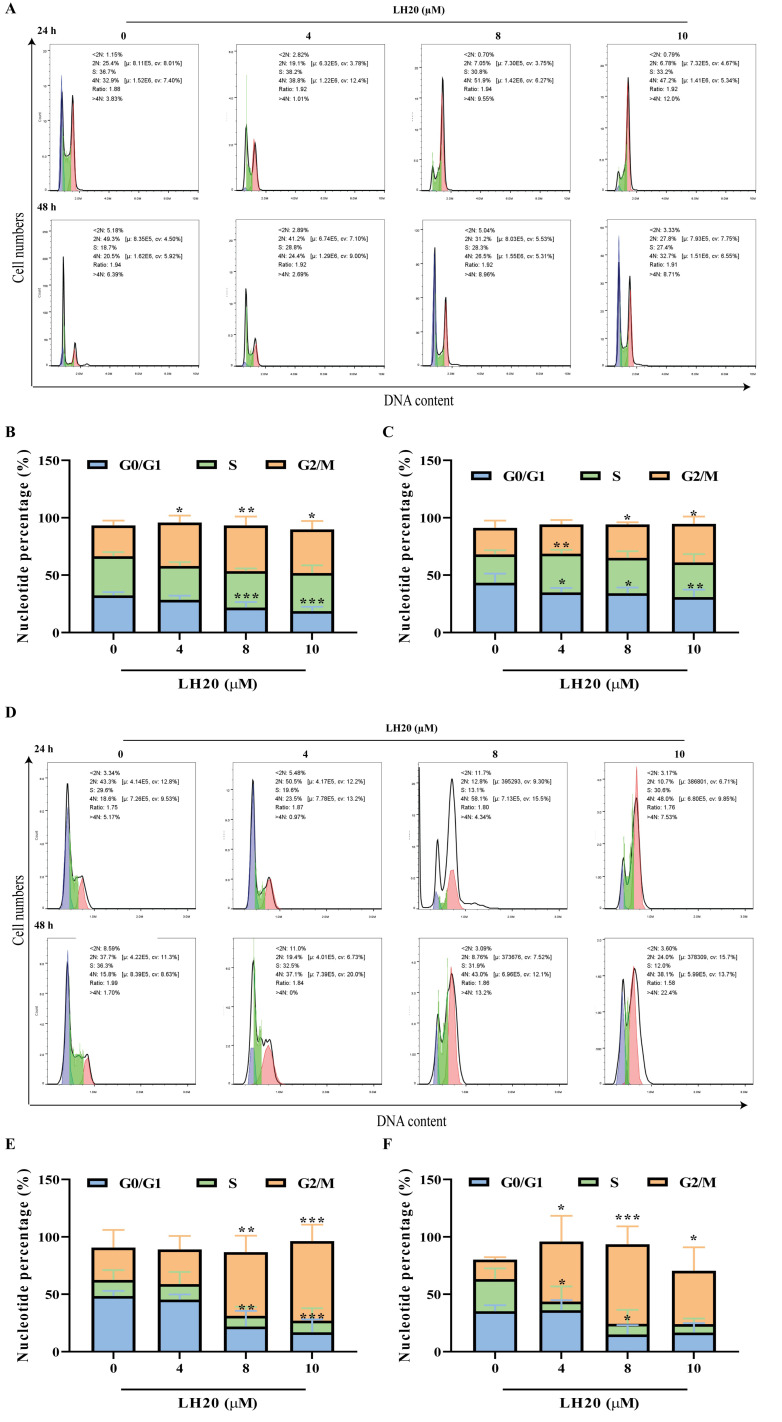
LH20 arrested G2/M phase of GBM cells. (**A**) Representative graphs from flow cytometry showing U87MG cells. Analysis of G0/G1, S, and G2/M phase rate of U87MG cells treated with LH20 for 24 h (**B**) and 48 h (**C**). (**D**) Representative graphs from flow cytometry showing U251 cells. Analysis of G0/G1, S, and G2/M phase rate of U251 cells treated with LH20 for 24 h (**E**) and 48 h (**F**). * *p* < 0.05, ** *p* < 0.01, *** *p* < 0.001 compared with the untreated group.

Treatment with LH20 at 4, 8, and 10 μM did not increase the G1 fraction of U87MG cells but promoted a gradual release of arrested cells from the G1/S checkpoint, thereby increasing the G2/M fraction and a corresponding reduction in the G1 fraction (Figure 5A–C). In particular, U251 cells treated with 8 and 10 μM LH20 for 24 and 48 h showed a fold increase in the nucleotide percentage (Figure 5D–F).

### 2.5. CDK4/6 Were Predicted to Be the Targets of LH20

**Figure 6:**
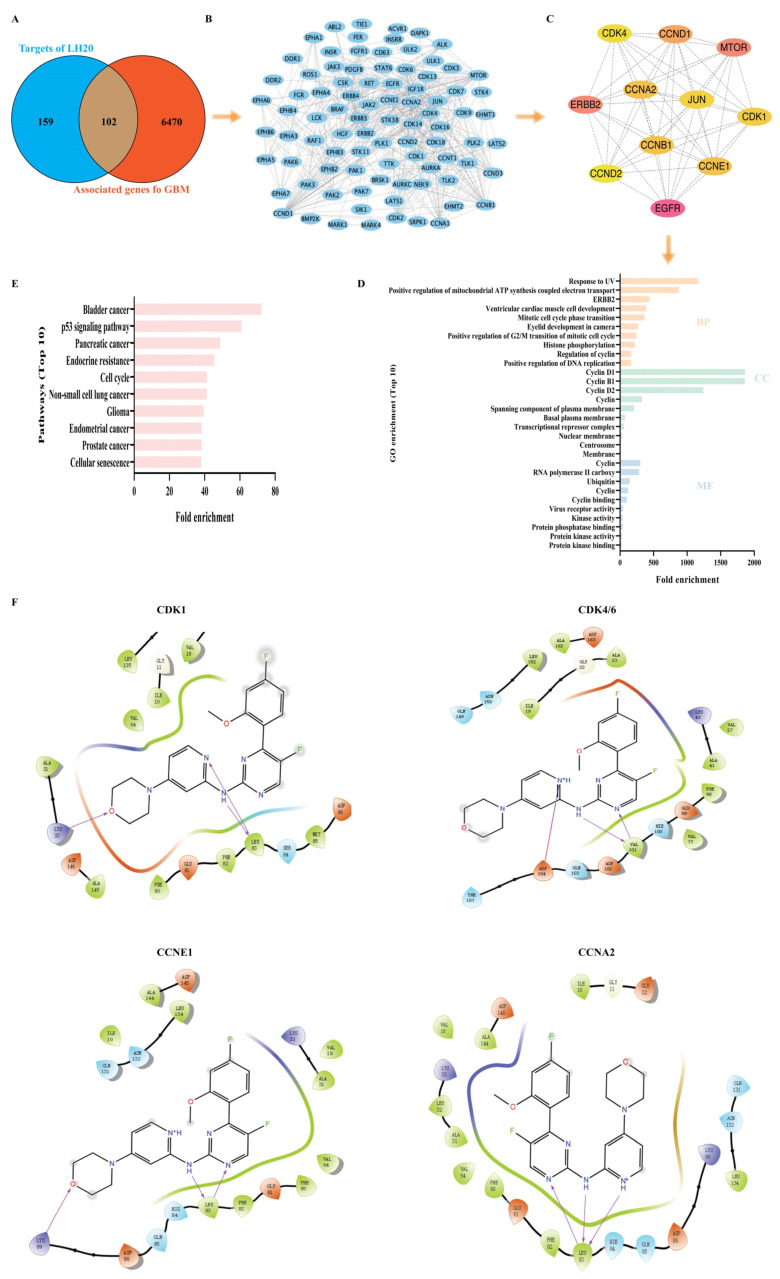
Predicted targets of LH20. (**A**) Venn graphs showing common genes between GBM and target genes of LH20. (**B**) Overlapped genes. (**C**) The 11 genes, nodes with a betweenness of connectivity varied from 129 to 900 were indicated in yellow and pink. (**D**) Gene ontology (GO) enrichment analysis of the hub genes. Biological process (BP), cellular component (CC), molecular function (MF). (**E**) Pathway analysis of the hub genes. (**F**) Molecular docking of LH20 and CDK1, CDK4/6, CCNE1, and CCNA2.

To elucidate the mechanism underlying the anti-GBM effects of LH20, we used the SwissTargetPredition database to predict the targets of LH20 and identified 102 target genes associated with GBM (Figure 6A,B). A total of 11 targets (CDK1, CDK4, JUN, CCNA2, CCND2, CCND1, CCNE1, CCNB1, EGFR, ERBB2, and MTOR) that met the criteria (median degree centrality (DC) ≥ 12.52, closeness centrality (BC) ≥ 117.43, and betweenness centrality (CC) ≥ 0.44) were defined as the core genes (Figure 6C). These core genes were enriched in response to UV light, positive regulation of mitochondrial ATP synthesis-coupled electron transport, ERBB2, ventricular cardiac muscle cell development, mitotic cell cycle phase transition, eyelid development in camera, positive regulation of G2/M transition of the mitotic cell cycle, histone phosphorylation, regulation of cyclin, and positive regulation of DNA replication, in terms of biological processes (Figure 6D). Pathway enrichment analysis revealed that bladder cancer, the p53 signaling pathway, pancreatic cancer, endocrine resistance, cell cycle, non-small cell lung cancer, glioma, endometrial cancer, prostate cancer, and senescence pathway were closely related to the mechanism of LH20 (*p* < 0.05). Further analysis using molecular docking confirmed that LH20 may mimic the adenosine of ATP and binds to the hinge regions of CDK1, CKD4/6, CCNE1, and CCNA2 to form several interactions within the binding pocket (Figure 6F).

### 2.6. LH20 Inactivates Phosphorylation of Retinoblastoma (Rb), the Downstream Protein of CDK4/6

**Figure 7:**
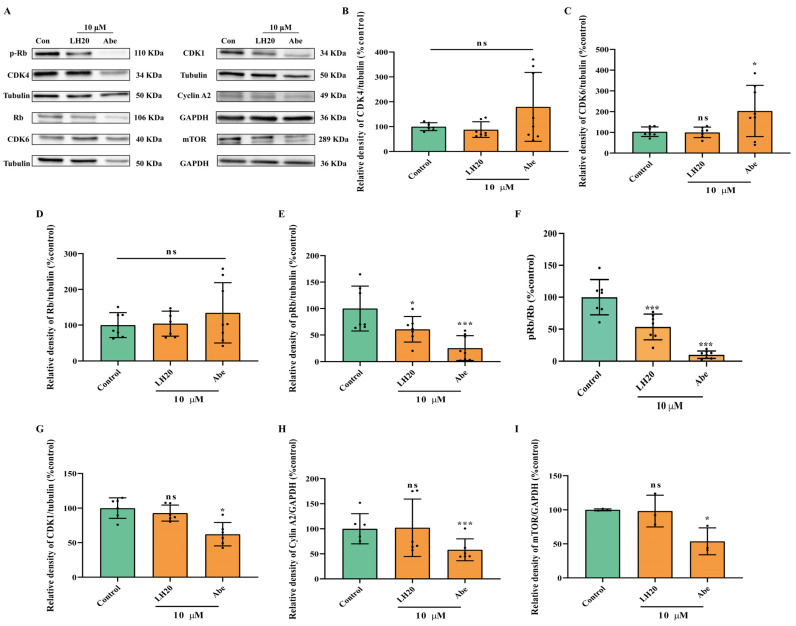
U87MG cells treated with LH20 for 24 h inactivated Rb phosphorylation. (**A**) Representative blots for CDK4, CDK6, Rb, p-Rb, CDK1, Cyclin A2, and mTOR. (**B**) Protein expression analysis of CDK4. (**C**) CDK6. (**D**) Rb. (**E**) pRb. (**F**) Ratio of pRb and Rb. (**G**) CDK1. (**H**) Cyclin A2. (**I**) mTOR. Data are expressed as mean ± standard deviation; ns represented no significance, * *p* < 0.05, *** *p* < 0.001 compared with the untreated group.

**Figure 8:**
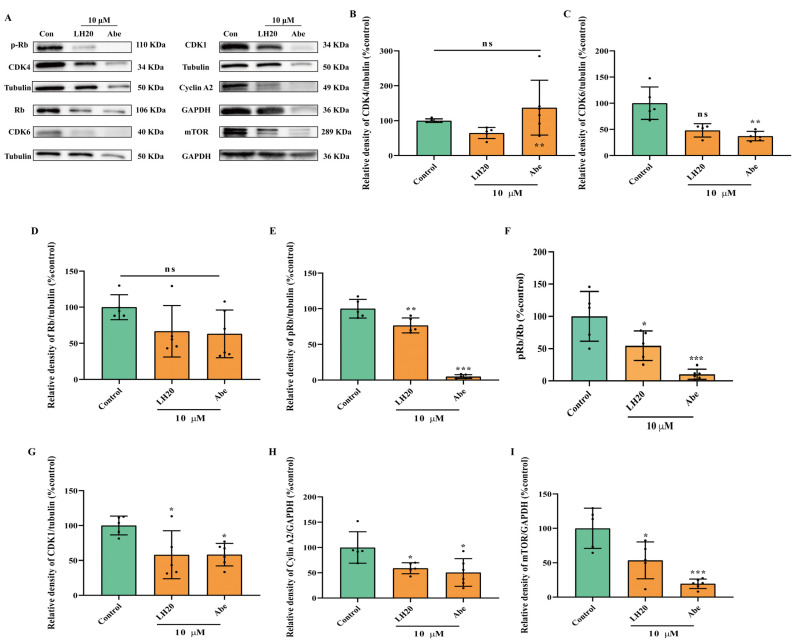
U87MG cells treated with LH20 for 48 h inactivated Rb phosphorylation. (**A**) Representative blots for CDK4, CDK6, Rb, p-Rb, CDK1, Cyclin A2, and mTOR. (**B**) Protein expression analysis of CDK4. (**C**) CDK6. (**D**) Rb. (**E**) pRb. (**F**) Ratio of pRb and Rb. (**G**) CDK1. (**H**) Cyclin A2. (**I**) mTOR. Data are expressed as mean ± standard deviation; ns represented no significance, * *p* < 0.05, ** *p* < 0.01, *** *p* < 0.001 compared with the untreated group.

Based on the predicted targets, we further detected the protein expression of CDK1, CDK4, and CDK6, and their downstream proteins, Rb, pRb, and mTOR. The results suggested that LH20 did not influence the protein expression of CDK4 and CDK6, but significantly decreased the phosphorylation level of Rb to 60.76 ± 24.14% (24 h, Figure 7) and 76.55 ± 10.56% (48 h, Figure 8). Moreover, U87MG cells treated with LH20 for 48 h showed lower expression of CDK1 (58.07 ± 34.34%), CyclinA2 (59.04 ± 10.91%), and mTOR (53.48 ± 26.86%), compared with that in untreated cells (100.00 ± 13.52%, 100.00 ± 30.97%, 100.00 ± 29.22%, respectively). This suggests that the mechanism of the anti-GBM effects of LH20 for 48 h exposure involves the inhibition of pRb, CDK1, CyclinA2, and mTOR.

## 3. Discussion

GBM is a brain tumor with the highest degree of malignancy and worst prognosis, and is highly prone to recurrence and metastasis. Currently, TMZ is the only therapeutic drug approved for GBM treatment; however, drug resistance often occurs during treatment, thereby limiting its outcomes. Therefore, there remains an urgent need to identify new drugs for the treatment of GBM. In this study, we identified LH20 as a new potential compound for the treatment of GBM using the U87MG and U251 cell lines.

The characteristics of this malignant tumor include uncontrolled cell proliferation, invasiveness, extensive angiogenesis, and the ability to alter multiple genetic genes [13]. LH20, a novel pyrimidin-2-amine compound, inhibited the proliferation of U87MG and U251 cells similar to the effects of Abe. Furthermore, long-term exposure (72 h) to LH20 inhibited the survival of U87MG at concentrations of 1, 10, 30, and 60 μM (77.09 ± 4.29%, 51.75 ± 10.36%, 41.23 ± 1.83%, 29.63 ± 4.03%), confirming its long-term anti-GBM effects. Migration is a peculiar feature that favors the aggressiveness and poor prognosis of glioblastoma [14]. Using a typical migration test, we demonstrated the anti-migratory effect of LH20 on U87MG and U251 cells, as the ability of cells to close scratches decreased with LH20 treatment.

Apoptosis is a key cell survival process; however, its inhibition promotes tumor cell survival [15]. In the present study, we demonstrated that LH20 induces a late-phase apoptosis, resulting in necrosis. In particular, the mitochondrial membrane potential of GBM cells significantly decreased following treatment with LH20, confirming that LH20 stimulated programmed cell death, similar to the effects of Abe [16]. Moreover, prolonged exposure to LH20 promoted a gradual release of arrested cells from the G1/S checkpoint, thereby increasing the G2-M fraction and corresponding reduction in the G1 fraction, which was also observed following treatment with ribociclib [17].

Cell cycle progression is mainly driven by CDKs. The main cell-cycle-associated CDKs are CDK1, CDK2, CDK4, and CDK6, which directly regulate the cell cycle progression [18]. In particular, CDK4/6 is a part of the core pathways frequently altered in GBM [19]. A recent study confirmed that CDK4 is the most frequently overexpressed gene in GBM brain tissues [20], which contributes to the degree of malignancy [21]. The most frequent alterations leading to the activation of the CDK4/6 pathway in GBM are co-deletions of the CDKN2A and CDKN2B loci (56%), amplification of CDK4 (14%), and deletion of RB1 (7.9%) [7]. In the present study, we identified 11 core genes, including CDK1, CDK4, JUN, CCNA2, CCND2, CCNE1, CCNB1, EGFR, ERBB2, and mTOR using website prediction and molecular docking. Using Western blotting, we confirmed that LH20 did not influence the protein expression of CDK1, CDK4, and CDK6 but decreased the phosphorylation of Rb, which is downstream of CDK4 or CDK6. Cyclin D/CDK4/6 complexes phosphorylate Rb, resulting in the release of E2F factors for Rb sequestration and cell cycle progression. In contrast, unphosphorylated Rb interacts with E2F transcription factors and inhibits E2F target gene expression and G1/S cell cycle transition [22]. Our molecular docking results showed that LH20 potentially binds to the hinge region of CDK4/6, thereby affecting Rb phosphorylation. However, treatment with LH20 for 48 h resulted in a lower expression of CDK1, CyclinA2, and mTOR, illustrating that LH20 could inhibit CDK1, CyclinA2, and mTOR for long-term exposure.

The predicted chemical properties suggest that LH20 has a molecular weight of 399.15, a topological polar surface area (tPSA) of 72.40 Å, and a partition coefficient (Log P) of 2.94. These properties allow LH20 to pass through the blood–brain barrier (BBB), as these predicted values approximate to “Egan-like” properties as follows: (1) MW< 450, (2) PSA < 79 Å, (3) LogP from +0.4 to 6.0 in SwissADME (http://www.swissadme.ch, accessed on 19 September 2022) [23], which suggest the potential BBB-penetrable ability of LH20 makes an excellent candidate for further development as a potential GBM treatment drug. However, the ability of LH20 to cross the BBB, which is a limitation of this study, requires further investigation. A virtual filter (Osiris Property Explorer: http://www.organic-chemistry.org/prog/peo/, accessed on 19 September 2022) [24] was applied to predict the cytotoxicities of LH20. LH20 was predicted to be safe and expected to display low or no toxicity, in terms of mutagenicity, tumorigenicity, irritant effects, and effects on the reproductive system. However, its cytotoxicity still needs further experimental studies using normal cells and mice, which is another limitation of this study.

## 4. Materials and Methods

### 4.1. Preparation of LH20

**Figure 9:**
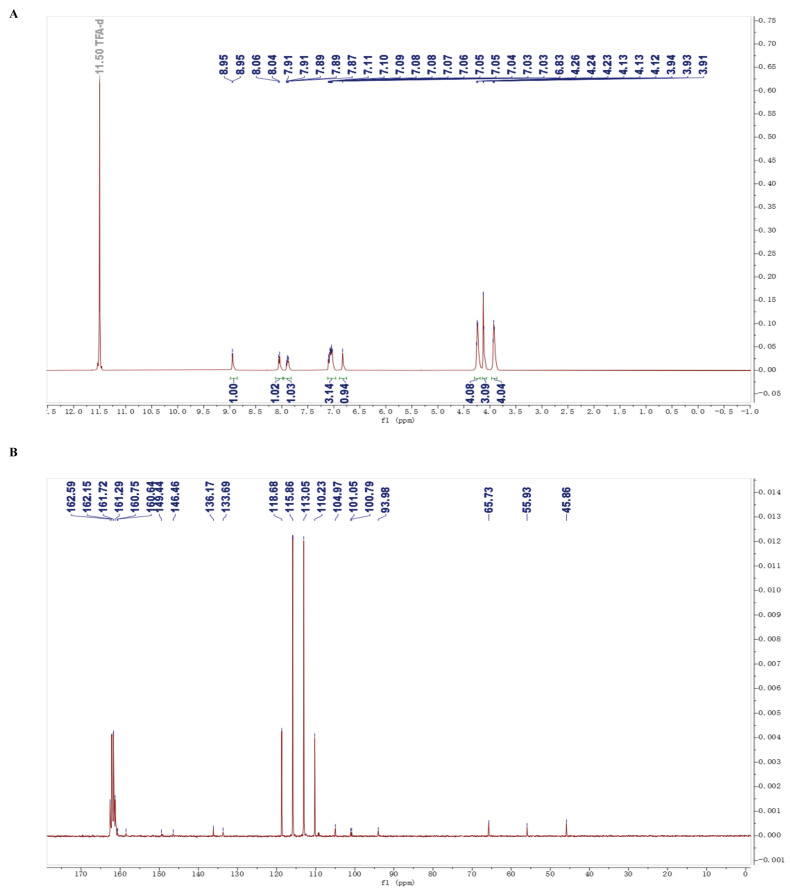
NMR spectra of LH20. (**A**) ^1^H NMR. (**B**) ^13^C NMR.

LH20 (98% purity, MW = 399.14) was synthesized as described previously [25], and a white solid powder was obtained. Briefly, the 2-chloro-5-fluoro-4-(4-fluoro-2-methoxyphenyl) pyrimidine intermediate was prepared using the Suzuki–Miyaura coupling reaction of commercial compounds (4-fluoro-2-methoxyphenyl) boronic acid and 2,4-dichloro-5-fluoropyrimidine with (dppf) Cl_2_ as the catalyst. This was followed by treatment with 4-morpholinopyridin-2-amine to afford the LH20 via the Buchwald–Hartwig amination reaction. The compound was tested using nuclear magnetic resonance (NMR, ECZ400S, JEOL, Tokyo, Japan) spectra. ^1^H NMR (400 MHz, CF_3_COOD) δ = 9.0 (d, J = 2.5, 1H), 8.1 (d, J = 7.4, 1H), 7.9–7.8 (m, 1H), 7.1 (ddt, J = 15.3, 7.8, 2.5, 3H), 6.8 (s, 1H), 4.2 (d, J = 4.9, 4H), 4.2–4.1 (m, 3H), 3.9 (d, J = 5.2, 4H). ^13^C NMR (100 MHz, CF_3_COOD) δ 160.75, 160.64, 158.49, 149.44, 146.46, 136.17, 133.69, 113.54, 109.36, 109.15, 104.97, 101.05, 100.79, 93.98, 65.73, 55.93, 45.86 (Figure 9). For cell experiments, a LH20 stock solution (20 mM) was prepared with DMSO (0.1%, Aladdin, Shanghai, China).

### 4.2. Cell Culture

U87MG and U251 cells were purchased from Procell (Wuhan, China) and cultivated in high glucose DMEM (Gibco, Carlsbad, CA, USA), appended by 10% fetal bovine serum (CLARK Bioscience, Richmond, VA, USA) and 1% antibiotics (streptomycin/penicillin, Biosharp, Hefei, Anhui, China) in a humidified incubator at 37 °C and 5% CO_2_.

### 4.3. Cell Viability

Cells were inoculated into 96-well microplates at a density of 100,000 cells/well with LH20 or Abe (CAS: 1231929-97-7, D&B Bioscience, Shanghai, China) for 24 or 48 h. After removing the culture medium, cells were incubated with refresh culture medium containing 20 µL Cell Counting Kit-8 (CCK8, Biosharp) reagent for 30 min at 37 °C. Cell proliferation was evaluated by measuring the absorbance (450 nm) using a microplate reader (Spectra MAX 190, Molecular Devices, San Jose, CA, USA), as previously described [26]. Survival rate was calculated using as follows:

survival rate = abs of LH20/abs of control × 100%.

### 4.4. LDH Assay

The level of LDH released from the U87MG cells was measured using a cytotoxic LDH assay (Nanjing Jiancheng Bioengineering Institute, Nanjing, China). Cells were seeded into 96-well plates at a density of 10,000 cells/well for 24 h and treated with 0, 0.1, 1, 4, 8, 10, 20, 100, and 200 µM of LH20 or Abe for 24 and 48 h, following which the medium was removed to determine the released content of LDH as previously described [27].

### 4.5. Wound Healing Assay

GBM cells were seeded in 6-well cell culture plates at a density of 100,000 cells/well and grown to 90% confluence. Confluent cell monolayers were scratched using a pipette tip and each well was washed with phosphate-buffered saline (PBS) to remove non-adherent cells. Then, the cells were treated with LH20 (4, 8, or 10 µM) and incubated for 24 or 48 h.

The perimeter of the central cell-free zone was determined using an optical microscope (Zeiss X-Cite) [28]. The migratory area was calculated by the formula: migratory area (%) = (area _[0 day]_ − area _[24 h/48 h]_)/area _[0 day]_ × 100%.

### 4.6. Annexin V-FITC/PI Apoptosis Assay

The cells treated with LH20 were harvested using trypsin (Biosharp, Hefei, Anhui, China) and centrifuged at 4 °C and 300× *g* for 5 min. Aliquots of 100,000 cells were suspended with 500 µL binding buffer and 5 µL staining reagent (Boster, Wuhan, China). After incubation in the dark at 37 °C for 5 min, the fluorescence intensities of FITC and PI were analyzed using a flow cytometer (Agilent NovoCyte, Santa Clara, CA, USA) [29].

### 4.7. Determination of MMP

Cells (U87MG and U251) were treated with 0, 4, 8, or 10 µM of LH20 for 24 or 48 h, then incubated with 500 µL of JC-1 working solution at 37 °C for 20 min, and centrifuged at 300× *g* for 5 min; the supernatant was discarded. The harvested cells were resuspended with 120 µL of JC-1 staining buffer and analyzed using a flow cytometer.

### 4.8. Cell Cycle Analysis

Cell cycle analysis was performed using a Cell Cycle Staining Kit (MULTISCIENCES, Hangzhou, Zhejiang, China) in a 24-well plate. Cells treated with 0, 4, 8, and 10 µM of LH20 were harvested with trypsin and washed with PBS. Cells were stained with 1 mL DNA staining solution and 10 µL of permeabilization solution at 37 °C for 30 min and analyzed using flow cytometry. The cell cycle was simulated using FlowJo software.

### 4.9. Predicting Targets and Pathways of LH20

The canonical SMILES of LH20 was retrieved from the ChemDraw Ultra 2017 software and used for target identification in the Swiss Target Prediction database (http://www.swisstargetprediction.ch, accessed on 19 September 2022). Target genes associated with glioblastoma were obtained from GeneCards (https://www.genecards.org/, version 4.9.0, accessed on 19 September 2022). Overlapping genes between the target of LH20 and glioblastoma were retrieved using EVenn (http://www.ehbio.com/test/venn/#/, accessed on 19 September 2022) [30], while the protein–protein interaction (PPI) network of the target was obtained using the STRING online tool (https://string-db.org/, accessed on 19 September 2022). Core genes were analyzed using CytoNCA (Cytoscape3.6.1 (https://www.cytoscape.org/, accessed on 19 September 2022) with the criteria of median DC, CC, and BC [31]. Gene ontology (GO) and biochemical pathways of the core targets were determined using the web-based annotation tool DAVID v6.8 (https://david.ncifcrf.gov/tools.jsp, accessed on 19 September 2022). Statistical significance was set at *p* < 0.05.

### 4.10. Molecular Docking

The crystallographic structures of CDK1, CDK4/6, CCNE1, and CCNA2 were downloaded from the Protein Data Bank (PDB codes: 6GU2, 2EUF, 7KJS, and 4BCP, respectively). Protein preparation was performed using the Protein Preparation Wizard and Receptor Grid Generation modules. Preparation of all LH20 was initially minimized by the LigPrep module with ionization-generated possible states at a target pH of 7.0 ± 2.0 s. For the force field option, OPLS4 and the other parameters were set to their default values. LH20 was flexibly docked into the ligand site of the co-crystal complex using the Ligand Docking module with standard settings in both standard precision (SP) modes. Only the best pose with good hydrogen bond geometries and low-energy conformations was considered for further analyses [25].

### 4.11. Western Blotting Analysis

Cells were seeded in DMEM with 10% FBS for 24 h and treated with LH20 (10 μM) and Abe (10 μM). Proteins were extracted from U87MG cells using Triton X-100 lysis buffer. Protein lysates were resolved using 10% SDS-PAGE (Beyotime, Shanghai, China) and transferred to PVDF (Millipore, Merck, NJ, USA) via electroblotting. Each blot was incubated for 15 min at room temperature in Quick block solution (Beyotime) and overnight at 4 °C with primary antibody (Table 1), diluted in bovine serum albumin (BSA). The membranes were incubated for 1 h at room temperature with horseradish peroxidase-conjugated secondary antibodies (Proteintech, Wuhan, Hubei, China). The blots were analyzed using a ChemiDoc XRS imaging system (Bio-Rad, Hercules, CA, USA), and the digital signals were quantified using ImageJ Software (1.52a, Wayne Rasband, Bethesda, MD, USA).

### 4.12. Statistical Analysis

Data are expressed as mean ± standard deviation (SD). Normal distribution of data was tested using the Shapiro–Wilk test, and normally distributed data were analyzed using one-way analysis of variance (ANOVA) with Dunn’s test for multiple groups. Otherwise, non-normally distributed data were analyzed using the Kruskal–Wallis test. Differences were considered significant at *p* < 0.05. Statistical analyses were performed, and figures were generated using GraphPad Prism (version 9.0.0).

## 5. Conclusions

We demonstrated that LH20 exhibited significant growth inhibitory and migratory effects against GBM cells in vitro, induced late-phase apoptosis and MMP drop, and arrested cells in the G2/M phase. Furthermore, the molecular mechanism illustrated that LH20 potentially targets CDK4/6 to inactivate Rb phosphorylation and influence mTOR expression. Overall, our study suggests LH20 as a novel treatment option for patients with GBM.

## Figures and Tables

**Table 1:** Information on antibodies.

Antibody	Source	Identifier	Dilution Rate
Rabbit anti-CDK1	Proteintech	Cat# 19532-1-AP	1:1000
Rabbit anti-CDK4	Abcam	Cat# ab199728	1:2000
Mouse anti-CDK6	Santa Cruz Biotechnology	Cat# sc-7961	1:200
Mouse anti-Rb	Santa Cruz Biotechnology	Cat# sc-102	1:200
Rabbit anti-pRb (Ser807/811)	Cell Signaling Technology	Cat# 8516	1:1000
Mouse anti-CylinA2	Proteintech	Cat# 66391-1-lg	1:5000
Rabbit anti-GAPDH	Proteintech	Cat# 10494-1-AP	1:6000
Mouse anti-Tubulin	Proteintech	Cat# 66031-1-lg	1:20,000

## Data Availability

The data presented in this study are available upon request from the corresponding author.

## Sample Availability

Samples of the compound are available from the authors.
